# Change in Mean Frequency of Resting-State Electroencephalography after Transcranial Direct Current Stimulation

**DOI:** 10.3389/fnhum.2016.00270

**Published:** 2016-06-06

**Authors:** Tjeerd W. Boonstra, Stevan Nikolin, Ann-Christin Meisener, Donel M. Martin, Colleen K. Loo

**Affiliations:** ^1^School of Psychiatry, University of New South WalesSydney, NSW, Australia; ^2^Black Dog Institute, University of New South WalesSydney, NSW, Australia; ^3^Institute of Cognitive Science, University of OsnabruckLower Saxony, Germany; ^4^Department of Psychiatry, St. George Hospital, South Eastern Sydney HealthSydney, NSW, Australia

**Keywords:** tDCS, DLPFC, healthy volunteer, cortical oscillations, EEG mean frequency

## Abstract

Transcranial direct current stimulation (tDCS) is proposed as a tool to investigate cognitive functioning in healthy people and as a treatment for various neuropathological disorders. However, the underlying cortical mechanisms remain poorly understood. We aim to investigate whether resting-state electroencephalography (EEG) can be used to monitor the effects of tDCS on cortical activity. To this end we tested whether the spectral content of ongoing EEG activity is significantly different after a single session of active tDCS compared to sham stimulation. Twenty participants were tested in a sham-controlled, randomized, crossover design. Resting-state EEG was acquired before, during and after active tDCS to the left dorsolateral prefrontal cortex (15 min of 2 mA tDCS) and sham stimulation. Electrodes with a diameter of 3.14 cm^2^ were used for EEG and tDCS. Partial least squares (PLS) analysis was used to examine differences in power spectral density (PSD) and the EEG mean frequency to quantify the slowing of EEG activity after stimulation. PLS revealed a significant increase in spectral power at frequencies below 15 Hz and a decrease at frequencies above 15 Hz after active tDCS (*P* = 0.001). The EEG mean frequency was significantly reduced after both active tDCS (*P* < 0.0005) and sham tDCS (*P* = 0.001), though the decrease in mean frequency was smaller after sham tDCS than after active tDCS (*P* = 0.073). Anodal tDCS of the left DLPFC using a high current density bi-frontal electrode montage resulted in general slowing of resting-state EEG. The similar findings observed following sham stimulation question whether the standard sham protocol is an appropriate control condition for tDCS.

## Introduction

Transcranial direct current stimulation (tDCS) is a non-invasive brain stimulation technique used to explore cognitive functioning across a wide range of domains (Coffman et al., 2014). Therapeutic potential has been reported in neuropathological disorders including depression (Arul-Anandam and Loo, 2009; Nitsche et al., 2009; Loo et al., 2010, 2012; Brunoni et al., 2013), stroke (Boggio et al., 2007; Baker et al., 2010), and dementia (Kuo et al., 2014). A low current, typically 1–2 mA, is applied across the brain through two or more electrodes placed on the head, modulating the activity of brain networks (Fregni and Pascual-Leone, 2007). Complex interactions of stimulation polarity, direction of electric current (i.e., radial or perpendicular to neuronal axes), and baseline levels of neuronal activity determine whether tDCS effects will be excitatory or inhibitory (Nitsche and Paulus, 2000; Jacobson et al., 2012b; Bikson and Rahman, 2013; Rahman et al., 2013). In the motor cortex, anodal stimulation partially depolarizes neuronal membranes and has been shown to result in increased excitability in regions underlying the electrode as quantified using motor-evoked potentials (Nitsche and Paulus, 2000). The after-effects of tDCS can last for an hour or longer following as little as 5 min of stimulation (Nitsche and Paulus, 2000).

Different imaging modalities such as functional magnetic resonance imaging (fMRI) and magnetic resonance spectroscopy (MRS) have been used to quantify the effects of tDCS to other brain regions (Keeser et al., 2011a; Hampstead and Gopinath, 2013; Rae et al., 2013). Electroencephalography (EEG) appears particularly promising as it can be used to also investigate how tDCS affects the cortical dynamics and hence brain functioning at a network level (Keeser et al., 2011b; Jacobson et al., 2012a; Spitoni et al., 2013; Accornero et al., 2014; Mangia et al., 2014; Powell et al., 2014; Romero Lauro et al., 2014). EEG has been used to investigate both the online effects of tDCS and offline effects by contrasting EEG activity before, during and after stimulation. The majority of prior studies have examined EEG activity following anodal (excitatory) tDCS stimulation (i.e., “offline” effects). Anodal tDCS to the right posterior parietal cortex resulted in a transient increase of alpha activity during the first 7.5 min following stimulation (Spitoni et al., 2013). Jacobson et al. (2012a) found a reduction of theta power in the region around the anode with stimulation of the right inferior frontal gyrus. A significant reduction in left frontal delta activity has been found following 20 min of 2 mA tDCS to the left dorsolateral prefrontal cortex (DLPFC; Keeser et al., 2011b). Twenty minutes of 1 mA anodal tDCS delivered to the same region, the left DLPFC, during an emotional valence modulation paradigm resulted in a significant decrease of alpha and an increase of beta-band activity after tDCS compared to pre-stimulation baseline (Maeoka et al., 2012). Together these findings suggest overall mixed effects, potentially due to heterogeneity in methodologies used—with differences in electrode montage, stimulus intensity, and time delay between stimulation and EEG recordings.

More recently, direct “online” effects of tDCS on EEG activity have also been examined using integrated systems which combine both modalities (Schestatsky et al., 2013). The stimulation artifacts of concurrent EEG appear limited: while some have reported an increase in broadband noise during tDCS (Soekadar et al., 2014), others have only found transient artifacts during the ramping phases of tDCS (Accornero et al., 2014; Romero Lauro et al., 2014). Results during “online” tDCS though have similarly been mixed. One study found that anodal tDCS over the left DLPFC resulted in an overall increase in the average frequency of brain activity, with a reduction in power at lower frequencies and/or an increase at higher frequencies (Accornero et al., 2014). In contrast, an increase in low frequency beta activity was reported during anodal stimulation of the left DLPFC using a bi-frontal (F3–F4) montage (Song et al., 2014). Similarly, Wirth et al. (2011) incorporated a language task during stimulation and EEG recording and found a significant reduction in delta power after anodal tDCS over the left DLPFC.

As such, the neuromodulatory effects of tDCS during and following stimulation measured using EEG remain unclear, due likely to differences in methodological approaches. The current study therefore sought to delineate the effects of tDCS on oscillatory resting-state activity. The left DLPFC was selected as the target for stimulation as this montage is commonly used for many different purposes, including the treatment of depression (Loo et al., 2010), cognitive enhancement of verbal memory (Nikolin et al., 2015), working memory (Mulquiney et al., 2011), cognitive training (Martin et al., 2013) and to reduce cravings (Boggio et al., 2009). We were also interested in investigating “online” effects during tDCS, given results from a prior study that cognitive training may be more effective when conducted during tDCS (Martin et al., 2014). To address this aim we used an integrated tDCS and EEG device (StarStim, Neuroelectrics, Spain), which has been specifically developed for concurrent tDCS and EEG (Schestatsky et al., 2013), to both investigate the direct effects on cortical oscillation during tDCS and the immediate after-effects of tDCS. This system uses small hybrid tDCS and EEG electrodes with a diameter of 3.14 cm^2^ for placement of multiple EEG electrodes as well as tDCS stimulating electrodes on the scalp. These electrodes are similar in size to those used for high-density tDCS (Nikolin et al., 2015) and would result in a higher current density compared to commonly used larger sponge electrodes (e.g., 35 cm^2^). Previous reviews have suggested that higher current densities are more effective at modulating brain activity (Bastani and Jaberzadeh, 2012; Hill et al., 2015). For this reason, we hypothesized pronounced effects on resting-state EEG with the use of smaller electrodes in this study. Integrated tDCS and EEG may offer a marker to robustly detect the neuromodulatory effects of tDCS.

## Materials and Methods

### Participants

Twenty healthy participants (9 females, mean age: 24.4 years, range 19–33) with no history of neurological or psychiatric disorders participated as paid volunteers in this study. The protocol was approved by the UNSW Human Research Ethics Committee (HC13278). All participants gave informed consent before participating in the experiment.

### Protocol

**Figure 1:**
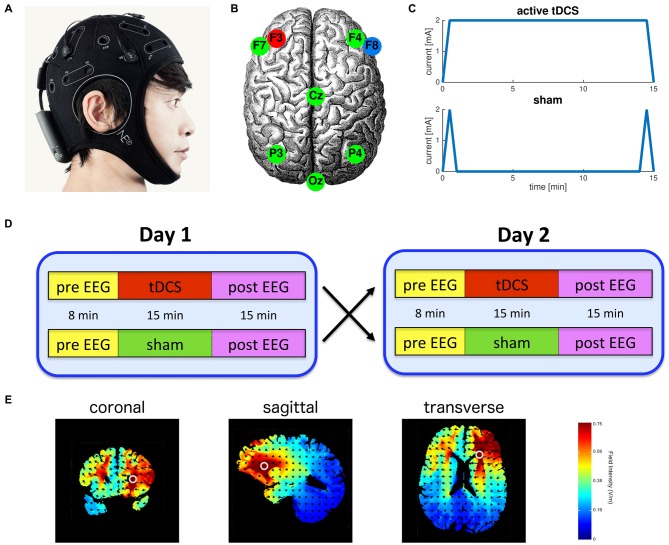
**Experimental protocol. (A)** Integrated transcranial direct current stimulation (tDCS) and electroencephalography (EEG) device (Starstim, Neuroelectrics Barcelona SL, Spain); **(B)** Anodal tDCS delivered to the left dorsolateral prefrontal cortex (anode: F3, cathode: F8) and the other six electrodes were used for EEG; **(C)** Electrical current waveforms associated with active tDCS and sham; **(D)** Participants were randomly assigned to receive either active or sham tDCS on the first session. All participants were fully crossed over to the other condition in the second session; **(E)** Model simulation using HDExplore^TM^ (Soterix Medical, New York, NY, USA) of the pattern of current strength associated with the bi-frontal tDCS montage used in this study.

Participants were tested in a sham-controlled, randomized, crossover study design. Active tDCS was applied for 15 min with an intensity of 2.0 mA (current density = 6.4 A·m^−2^), initiated by a 30 s ramp up of the current and terminated by a 30 s ramp down. In the sham condition the current was similarly ramped up over 30 s and immediately ramped back down over 30 s at the start and end of stimulation producing transient paraesthesia (e.g., itching and tingling) similar to sensations elicited by active tDCS and thereby effectively blinding the participant (Palm et al., 2013; Figure 1C). EEG was recorded for 8 min before stimulation to obtain baseline resting-state activity, during active or sham tDCS (15 min), and for 15 min post-stimulation (Figure 1D). During these recordings subjects were asked to keep their eyes open and fixated on a small target displayed on a computer screen.

### Data Acquisition

We used the 8-electrode StarStim system (Neuroelectrics Barcelona SL, Spain) to deliver tDCS and record EEG. Anodal tDCS was applied to channel F3 located over the left DLPFC with a 3.14 cm^2^ Ag/AgCl “Pistim” gel electrode. An identical return cathode was placed at channel F8 located above the right fronto-orbital region. The other six electrodes were used for EEG recording only and were located at F4, F7, Cz, P3, P4 and Oz (Figure 1B). These locations of the electrodes are specified by the standard 10–20 system. Pre- and post-stimulation EEG was recorded using all eight electrodes, while during stimulation EEG was recorded only using the six remaining electrodes not required for stimulation. All data were referenced against an electrode on the left earlobe and sampled at 500 Hz.

A short questionnaire was used to assess the self-reported rating of the physiological state of the participants at the beginning and end of each recording session. The questionnaire consisted of four questions using an 11-point Likert scale. The questions asked participants to rate their current physiological state compared to their usual level at the same time of day. The questions rated sleepiness (−5 much more sleepy to 5 much more awake), alertness (−5 much more distracted to 5 much more alert), vigor (−5 much less energetic to 5 much more energetic), and confusion (−5 much more confused to 5 much more clear minded). The questionnaire was administered directly before the pre EEG and directly after the post EEG on both the day of active tDCS and the day of sham tDCS.

### Spectral Analysis

Effects of tDCS on ongoing brain activity were characterized as the change in oscillatory EEG activity against baseline, which we tested using three contrasts: (1) post active—pre active; (2) post sham—pre sham; and (3) (post active—pre active) – (post sham—pre sham). Changes in oscillatory activity are thought to reflect changes in the local synchronization of cortical populations (Pfurtscheller and Lopes da Silva, 1999), and have been widely used to assess the effect of brain stimulation on cortical activity (Keeser et al., 2011b; Jacobson et al., 2012a; Spitoni et al., 2013; Mangia et al., 2014; Powell et al., 2014). EEG data was filtered using a Butterworth zero-phase band-pass filter (0.5–70 Hz) and a notch filter at 50 Hz to remove line noise. Data was segmented in 1.5 s intervals and segments containing eye, heart or muscle artifacts were semi-automatically identified. To identify segments containing artifacts, the amplitude of the signals were calculated (the Hilbert envelope) and *z*-transformed based on the mean and standard deviation across all samples. The *z*-transformed data was averaged across channels and segments containing samples that exceeded the threshold were rejected (cf., Oostenveld et al., 2011). The remaining segments were windowed using a Hanning window and the power spectral density (PSD) was computed using the Welch (1967) method. PSD were log-transformed before computing the changes in spectral power of EEG activity recorded pre and post the brain stimulation intervention.

### Partial Least Squares

We used PLS to investigate significant changes in EEG power after tDCS. PLS is a multivariate statistical technique that finds a linear regression model by projecting the independent and dependent variables to a new space, which is rank ordered by the percent covariance explained. That is, it decomposes the original data into orthogonal modes that account for the part of the covariance structure that correlates with a specified contrast (McIntosh et al., 2004; Langdon et al., 2011; Boonstra et al., 2013). We evaluated the following contrast using PLS: (1) post active—pre active; (2) post sham—pre sham; and (3) (post active—pre active)—(post sham—pre sham). The PSD of each EEG channel were the dependent variables; these consisted of 46 frequency bins and 8 channels for each participant. After applying the contrast, the data is averaged across participants and the resulting 46 × 8 matrix is decomposed into orthogonal components consisting of an eigenvalue, the latent variable (here frequency spectra consisting of 46 bins) and the corresponding weights for each channel (8 channels). As we used three contrasts, we performed three separate tests and no correction for multiple comparisons is required.

We generated surrogate data using permutation testing to determine which PLS components were statistically significant (*P* < 0.05; McIntosh et al., 2004; Langdon et al., 2011). For each subject, we randomly permuted the power spectra for pre and post stimulation before regressing the data against the contrast to obtain subject-level surrogate data (46 × 8 matrix). Similar to the original data, these surrogate data were averaged across all 20 participants and decomposed into orthogonal components. This analysis was performed for 1000 realizations to estimate the distribution of surrogate components. These surrogate components embody the expected distribution of values under the null hypothesis that there is no effect of brain stimulation (and hence pre- and post-stimulation data or active and sham data are exchangeable). PLS components were considered statistically significant if their eigenvalue exceeded 95% of the corresponding eigenvalues of this surrogate distribution (*P* < 0.05). The significance of the EEG channel and their spectral contents was then examined using bootstrapping (McIntosh et al., 2004; Langdon et al., 2011). For bootstrapping the same contrast is used as for the original analysis, but now the 46 × 8 matrix is averaged across a random sample (with replacement) of all participants. The grand average is decomposed into orthogonal components and this is again repeated for 1000 realizations. The obtained surrogate distribution can then be used to assess the between-subject variability and hence the confidence intervals of the extracted latent variables and weights (Efron and Tibshirani, 1986).

### EEG Mean Frequency

We also investigated changes in EEG mean frequency induced by tDCS. The mean frequency is used as an indicator of general slowing of EEG activity (Salinsky et al., 1991; Pop-Jordanova and Pop-Jordanov, 2005; Accornero et al., 2014). To compute the mean frequency we first normalized the PSD to the total power: $PSD_{norm}\left(i\right)\;=\;PSD(i)/\text{​}\sum_{i}PSD(i).$ The mean frequency was then defined as

(1) $$mf\;=\;\sum_{i}f(i)PSD_{norm}(i),$$

where, the index *i* denotes the frequency bin, *f(i)* the mean frequency in Hz for each frequency bin, and *PSD_norm_(i)* the relative power in that frequency bin. Because we used 1.5-s windows for the spectral decomposition, the frequency resolution (width of frequency bin) is 0.667 Hz. For the calculation of the mean frequency, we used the PSD on a frequency interval of 0–100 Hz. We averaged the mean frequency across all eight EEG channels to obtain a scalar value in each condition and for each participant. To investigate how the mean EEG frequency changes over time following tDCS, we segmented EEG traces post tDCS into three segments of 5 min each (0–5, 5–10 and 10–15 min post tDCS) and determined the mean EEG frequency for each segment separately.

### Statistical Analysis

Paired *t*-tests were used to statistically compare EEG mean frequency between pre and post and between active tDCS and sham. Wilcoxon signed-rank test was used to compare the subjective ratings between pre and post and between active tDCS and sham. Spearman’s rank correlation coefficient was used to quantify the relationship between the change in subjective rating and the change in EEG mean frequency. The difference scores were computed as post minus pre. To investigate the temporal effects of tDCS, we compared the change in mean EEG frequency at the three time segments (0–5, 5–10 and 10–15 min post tDCS) using 3 × 2 repeated measures ANOVA. Effects were considered statistically significant at *P* < 0.05.

## Results

### EEG Signals During and Following tDCS

**Figure 2:**
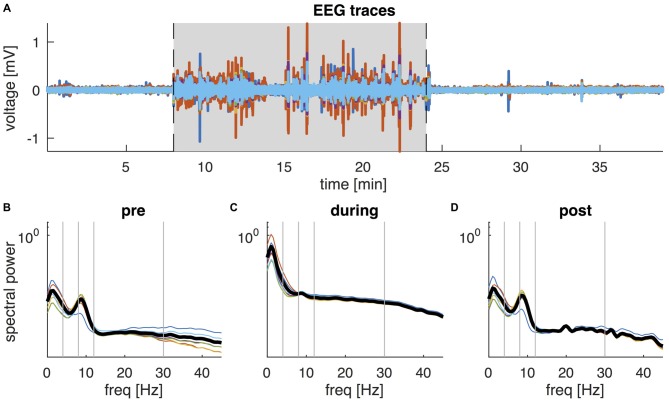
**EEG data of a representative participant. (A)** Butterfly plot of the six EEG channels (F7, F4, Cz, P3, P4, Oz) during the 38-min recording. Shaded area shows the interval of active tDCS. **(B–D)** Shows the corresponding power spectral density (PSD) of the six EEG channels during the interval before tDCS **(B)**, during tDCS **(C)**, and following tDCS **(D)**. Thick black lines show the average PSD across channels. Vertical lines separate the frequencies into the conventional frequency bands: delta (0–4 Hz), theta (4–8 Hz), alpha (8–12 Hz), beta (13–30 Hz) and lower gamma (30–45 Hz).

The characteristics of EEG activity recorded during tDCS changed markedly. In 8 of the 18 participants the EEG amplitude increased dramatically for the duration of tDCS, whereas in the other participants the increase in amplitude was mainly confined to the beginning and end of stimulation. Figure 2 shows data from a representative subject, showing an order of magnitude increase in EEG amplitude. The corresponding PSD revealed a broad increase in spectral power across all frequencies, although the largest increase was observed at frequencies below 5 Hz. Moreover, while a clear alpha peak can be observed in the EEG recordings pre and post tDCS, this peak is no longer discernible during tDCS but is replaced by broadband noise across all frequencies. More intermittent high amplitude fluctuations were observed in other participants. These large increases in EEG power are not observed in normal EEG and most likely reflect stimulation artifacts. Given the extent of these artifacts, we decided not to use the EEG data recorded during tDCS for further analysis.

**Figure 3:**
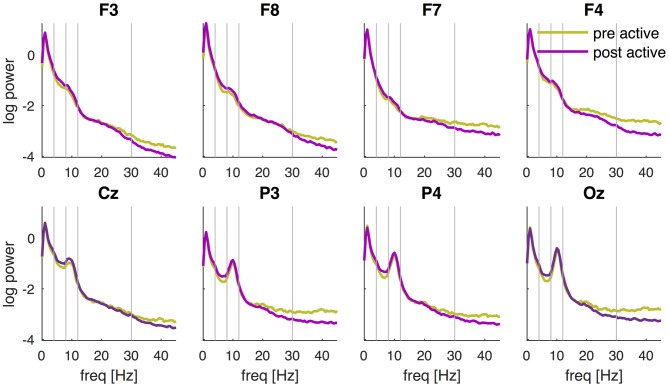
**PSD pre and post active tDCS.** PSD is determined during the resting-state recording pre and post tDCS and averaged across participants for each EEG channel (F3, F8, F7, F4, Cz, P3, P4 and Oz) separately. Power is displayed on a logarithmic scale. Vertical lines separate the frequencies into the conventional frequency bands: delta (0–4 Hz), theta (4–8 Hz), alpha (8–12 Hz), beta (13–30 Hz) and lower gamma (30–45 Hz).

**Figure 4:**
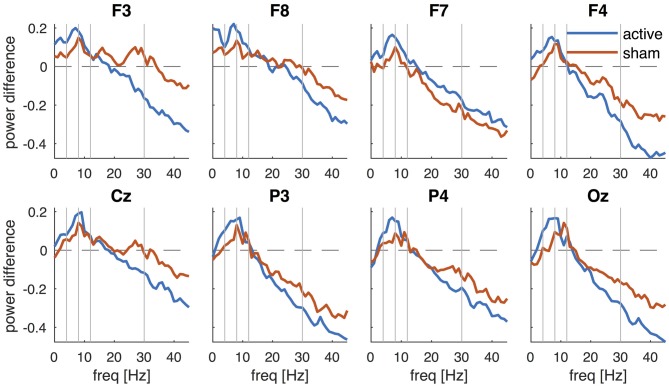
**Change in PSD after active tDCS and sham.** PSD_post_ is contrasted against PSD_pre_ and averaged across participants for each EEG channel (F3, F8, F7, F4, Cz, P3, P4 and Oz) separately. Vertical lines separate the frequencies into the conventional frequency bands: delta (0–4 Hz), theta (4–8 Hz), alpha (8–12 Hz), beta (13–30 Hz) and lower gamma (30–45 Hz).

The PSD of the EEG recorded before and after tDCS revealed a characteristic 1/f distribution, i.e., the power is inversely proportional to the frequency of the signal (Figure 3). In addition to the 1/f distribution, a peak in the alpha-band (8–12 Hz) can be observed which is most pronounced over posterior channels and maximal in channel Oz. Although the PSDs pre and post active tDCS revealed the same overall characteristics, some systematic differences were observed: the power at higher frequencies (>20 Hz) was reduced, while power around 8 Hz was increased after tDCS compared to the pre-tDCS baseline (cf., Figure 4). These changes in spectral power appeared similar across EEG channels.

To better reveal the changes in PSD after stimulation, Figure 4 shows the difference in log power (post-pre). After active tDCS, spectral power indeed decreased at higher frequencies and this decrease became larger at higher frequencies (Figure 4, blue lines). An increase in power was observed in the theta (4–8 Hz) and alpha bands with the largest increase at 7–9 Hz. The changes in PSD after sham revealed a similar frequency profile (Figure 4, red lines). Power was reduced at higher frequencies and increased at lower frequencies after sham, although the changes appeared smaller than those observed after active tDCS.

### Partial Least Squares

**Figure 5:**
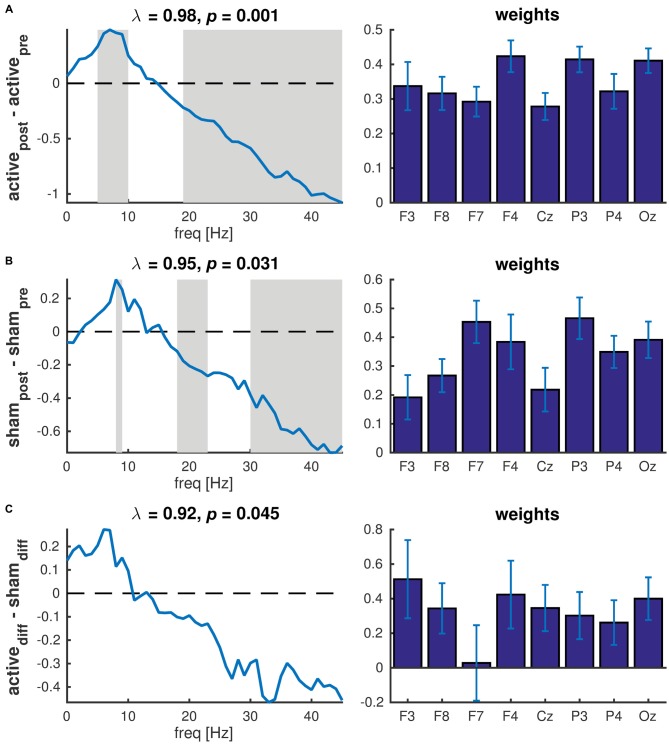
**Partial least squares (PLS) of PSD across all EEG channels. (A)** Contrast between pre and post active tDCS; **(B)** Contrast between pre and post sham; **(C)** Contrast between difference in active tDCS and sham. Left panels show the latent variables (frequency spectra) of each significant component revealing the changes in spectral power between conditions. The gray patches show the frequencies at which the difference was statistically significant as determined used bootstrapping. λ indicates the explained variance and *p* the *p*-value determined using permutation testing. Right panels show the corresponding weights reflecting the contribution of each EEG channel to the significant component. Error bars reflect the standard deviation estimated using bootstrapping.

We then used PLS to test whether these changes in PSD were statistically significant. PLS is a multivariate regression technique and a single regression model is constructed across all variables, rather than repeating the analysis for each variable, hence avoiding the problem of multiple comparisons. To test for changes in PSD after active tDCS, we used a simple contrast between pre and post and decomposed the resulting matrix. Permutation testing revealed one component that was statistically significant (*P* = 0.001). The latent variable revealed an increase in power at frequencies below 15 Hz and a decrease at frequencies above 15 Hz (Figure 5A, left panel). Bootstrapping revealed that the increase was significant (*P* < 0.05) at frequencies between 5 and 10 Hz with a peak at 7 Hz. The decrease in power was significant at frequencies above 19 Hz. The corresponding weights (Figure 5B, right panel) revealed a similar effect across all EEG channels. When repeating the same analysis for sham, one significant component was observed (*P* = 0.031). This component revealed a significant increase in power at 8–9 Hz and significant decrease at 18–23 Hz and above 30 Hz. Again, the effect was similar across EEG channels (Figure 5B).

To test whether the changes after tDCS were significantly different to the changes after sham, we contrasted the differences in PSD (post-pre) between active and sham. One component was significant (*P* = 0.045) and revealed an increase in power below 10 Hz and a decrease above 10 Hz in active tDCS compared to sham. However, bootstrapping showed that none of the individual frequency bins were significantly different. Although the effect was largely similar across channels, no significant difference was observed in channel F7 (Figure 5C).

### Mean EEG Frequency

**Figure 6:**
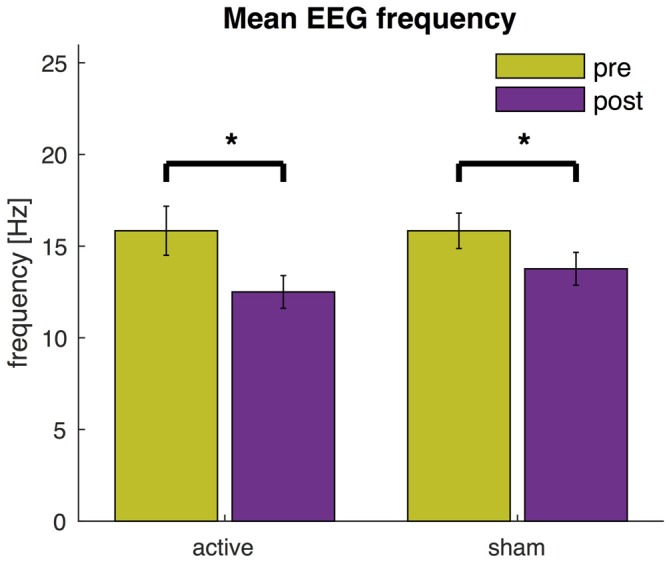
**EEG mean frequency before and after active tDCS and sham.** The mean frequency is averaged across all EEG channels. Error bars indicate the standard error and asterisks changes that are statistically significant (*P* < 0.05).

These multivariate analyses revealed a significant decrease in power at high frequencies and an increase at lower frequencies across all channels. We then used the mean EEG frequencies as a global indicator to quantify the slowing of EEG activity (Figure 6). After active tDCS, the mean frequency significantly reduced from 15.8 ± 1.3 Hz to 12.5 ± 0.9 Hz (*t*_(17)_ = 4.8, *P* < 0.0005). The mean frequency also decreased significantly after sham from 15.8 ± 1.0 Hz to 13.8 ± 0.9 Hz (*t*_(17)_ = 4.0, *P* = 0.001). The mean frequency after active tDCS (12.5 ± 0.9 Hz) was slightly lower than after sham (13.8 ± 0.9 Hz), but the difference was just above the significance threshold (*t*_(17)_ = −1.9, *P* = 0.073).

**Figure 7:**
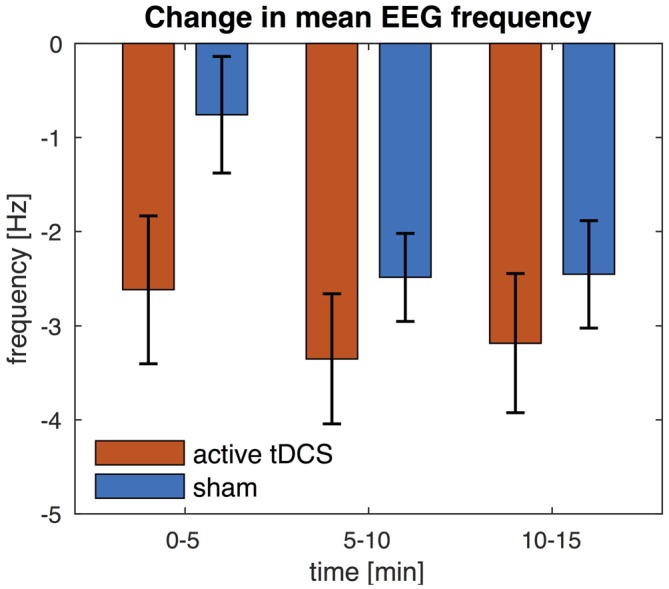
**Change in EEG mean frequency over time.** The mean frequency is averaged across all EEG channels. Change in mean frequency is shown for three separate time intervals (0–5, 5–10 and 10–15 min) and relative to pre-stimulation baseline. Error bars indicate the standard error.

We also compared the mean EEG frequency at three different time points (0–5, 5–10 and 10–15 min post tDCS). These results show that, while the mean EEG frequency was lower compared to pre tDCS baseline, the mean EEG frequency further decreased over time and the effect was greater after 5 min from the end of the stimulation than immediately after (see Figure 7). Indeed an ANOVA revealed a main effect of time (*F*_(2,34)_ = 17.5, *P* < 0.005). *Post hoc* *t*-test showed that the mean EEG frequency was significantly lower at 5–10 min (*t*_(17)_ = 4.4, *P* < 0.005) and 10–15 min (*t*_(17)_ = 5.1, *P* < 0.005) compared to 0–5 min. The main effect of condition and the interaction effect were not statistically significant (*P* > 0.05).

### Subjective Ratings of Physiological State

**Figure 8:**
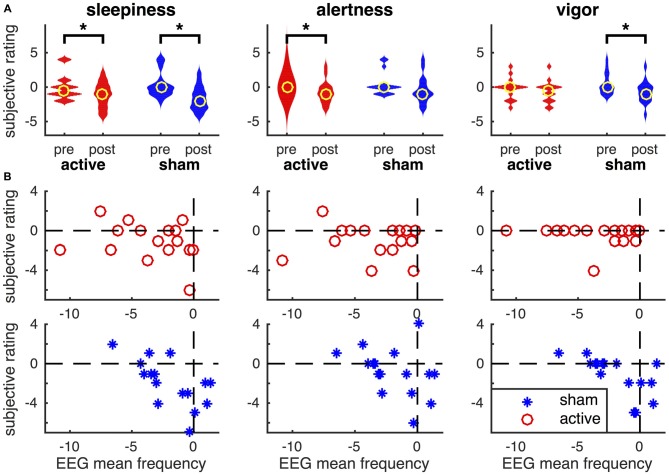
**Relationship between subjective ratings of their physiological state and the EEG mean frequency. (A)** Violin plot of subjective ratings of sleepiness, alertness and vigor before and after active tDCS and sham. Note, that polarity of sleepiness is reversed such that a higher rating means one is less sleepy. Yellow circles depict the median across participants and asterisks changes that are statistically significant (*P* < 0.05). **(B)** Relationship between changes in EEG mean frequency (on *x*-axis) and changes in subjective ratings of participant’s physiological state (*y*-axis). Changes are computed as post minus pre. Data for each participant are shown as red circles in active tDCS and blue asterisks for sham tDCS.

The subjective ratings of participant’s physiological state were compared pre and post tDCS (Figure 8A). Wilcoxon signed-ranks test (*n* = 16) indicated an increase in self-reported sleepiness after active tDCS (*P* = 0.034) and after sham (*P* = 0.008). Reported sleepiness was not significantly different after active tDCS compared to after sham. Alertness was reduced after active tDCS (*P* = 0.037), but was not significantly different after sham or between sham and active tDCS. Vigor was significantly reduced after sham (*P* = 0.027), but not after active tDCS and was also not significantly different after active compared to sham. No significant changes in confusion were found (not shown in Figure 8). We then investigated the relationship between these changes in subjective ratings and changes in EEG mean frequency using Spearman’s rank correlation coefficient (Figure 8B). This showed a significant correlation between the change in EEG mean frequency and the changes in sleepiness (rho = −0.71, *P* = 0.0018), alertness (rho = −0.51, *P* = 0.04), and vigor (rho = −0.71, *P* = 0.0019) in the sham condition. The negative correlations reveal that participants how show the largest reduction in EEG mean frequency reported the smallest reduction in subjective arousal. None of these relationships were statistically significant in active tDCS (*P* > 0.3).

## Discussion

The “online” and “offline” effects of bi-frontal tDCS on cortical oscillations were investigated using resting-state EEG in a sham-controlled randomized crossover study. EEG activity during tDCS revealed an extensive increase in broadband noise and was not further analyzed, as we could not uniquely distinguish stimulation artifacts from genuine changes in cortical activity. Results showed a significant increase in spectral power after tDCS compared to pre-stimulation baseline in the theta and alpha bands (with a maximum increase at 7 Hz) and a decrease in power at frequencies above 20 Hz across all EEG channels. A similar significant change in spectral power was observed after sham, although the change was of lesser magnitude as confirmed by a significant difference between active tDCS and sham. The latent variable extracted by PLS further revealed that the decrease in spectral power was almost linearly related to frequency, suggesting a slowing of resting-state EEG after tDCS. The EEG mean frequency—an indicator of slowing of EEG activity—was significantly reduced after both active tDCS and sham. The decrease in mean frequency was larger after active tDCS compared to sham, although this difference was just above the significance threshold (*P* = 0.073). These findings indicate a general slowing of cortical oscillations after anodal tDCS administered to the left DLPFC. “Sham” stimulation, consisting of a brief ramp up and down of current over a 1-min interval, also resulted in significant slowing of cortical activity.

Our finding of generalized slowing following anodal and sham tDCS of the left DLPFC appears to deviate from the pattern observed in previous studies. Using a similar bi-frontal montage and experimental paradigm, Accornero et al. (2014) noted a significant increase in mean frequency following active tDCS, and no change from baseline subsequent to sham stimulation. Similarly, other studies examining the EEG outcomes of tDCS to the prefrontal cortex report an increase in beta (Song et al., 2014), decrease in theta (Jacobson et al., 2012a), or a reduction in delta activity (Keeser et al., 2011b; Wirth et al., 2011). Although not explicitly analyzed, these findings would likely manifest in an increased mean frequency. However, the current study differs in several ways from these previous studies, which may account for the discrepancy between past experiments and present findings. A key difference in the present study is the use of smaller electrodes (3.14 cm^2^) with a 2 mA current, resulting in a maximum electric field magnitude of ~7.5 V/m (see Figure 1), compared to ~3.0 V/m for the montage used in the Accornero et al. (2014); field strength was estimated using HDExplore^TM^, Soterix Medical, New York, NY, USA. Recently, evaluation of motor cortical excitability changes following tDCS suggested that larger electrodes (35 cm^2^), with a corresponding reduced current density, produced a greater motor response compared with smaller electrodes (16 cm^2^; Ho et al., 2016). Although the relationship between current density and neurophysiological effects as measured by EEG remains unclear, recent evidence suggests a non-linear association between stimulation intensities and the direction of the resulting after-effects (Batsikadze et al., 2013). In addition, the effects of tDCS on executive function are also modulated by dopamine concentration in a non-linear manner, hinting at an inverted-U shaped dose response relationship (Plewnia et al., 2013; Nieratschker et al., 2015). Another difference is that the current study used eyes open resting state to avoid participants from falling asleep. In contrast, Keeser et al. (2011b) and Jacobson et al. (2012a) used eyes closed resting state, while Wirth et al. (2011) delivered tDCS while participants performed a task. This methodological difference may have affected outcomes, as the aftereffects of tDCS have previously been shown to depend on the physiological state during tDCS administration (Antal et al., 2007). In addition, differences in the effects of tDCS may also result from variability in anatomy (Seibt et al., 2015). Finally, in the current study, we used the StarStim system for integrated tDCS and EEG and further studies are required to determine whether differences in hardware features may result in variable effects of tDCS.

Unexpectedly, we also observed significant slowing of EEG activity following sham stimulation. Increases in power to low frequency oscillations have been documented in association with low cognitive engagement (Van Someren et al., 2011), and likely reflect states of low arousal such as drowsiness and fatigue (Boonstra et al., 2007). Participants in the current study were instructed to remain at rest for 38 min, and were thus likely to be experiencing the aforementioned states of reduced arousal during post-tDCS EEG recordings. Self-reports collected following completion of the EEG protocol seem to support this interpretation. At a group level, participants exhibited a decreased mean frequency in both active and sham conditions, which was accompanied by a similar reduction in certain subscales of subjective arousal (see Figure 8A). However, further analysis revealed that, at the individual level, there was an inverse correlation with greater reduction in mean EEG frequency associated with less sleepiness, more alertness and vigor in the sham condition only. Absence of such a correlation in the active group and effects on mean EEG frequency and subject ratings suggest that tDCS directly affected both the mean EEG frequency and subjective arousal, but with a decoupling of these outcomes.

Effects of sham stimulation were similar to those of active tDCS, although less pronounced. The slowing of resting-state EEG following sham stimulation is most likely related to changes in arousal and future studies should hence seek to maintain constant physiological state by restricting the duration of EEG recordings or including tasks to sustain constant engagement. However, if there is indeed a non-linear relationship between stimulation intensities and the direction of the resulting after-effects (Batsikadze et al., 2013), we should also consider the possibility of inducing measureable changes in cortical activity from brief stimulation (consisting of 60 mC of charge over the ramp up and down period) in the sham condition. This may raise questions regarding whether the “standard” ramp-up/ramp-down sham protocol is appropriate for EEG research. For example, Bastani and Jaberzadeh (2013) tested the effects of four different intensities of anodal tDCS to the left motor cortex on corticospinal excitability and found the lowest intensity (0.3 mA, with a total session charge of 180 mC) produced the largest changes in excitability. Further research is therefore warranted to identify potential effects of very low intensities of tDCS commonly applied for sham protocols. In particular, future studies should apply tDCS at multiple intensities (including no stimulation) to estimate the dose-response curve.

Analysis of EEG data recorded during tDCS revealed significant artifacts, similar to those described by Soekadar et al. (2014). These artifacts were judged to be too extensive for correction using data cleaning techniques and further analysis was thus not performed (see Figure 2A), in particular as these artifacts directly affect the spectral power of EEG. Previous studies of concomitant tDCS and EEG have had success using independent component analysis (ICA) to remove artifacts offline (Faria et al., 2012; Roy et al., 2014). These studies further report that noise resulting from stimulation was localized to EEG channels in close proximity to tDCS electrode locations (Accornero et al., 2014; Roy et al., 2014), and restricted to transient artifacts observed during the ramping phase (Accornero et al., 2014; Romero Lauro et al., 2014). However, the results of ICA are meaningful only when the amount of data and number of channels are large enough (Jung et al., 1998), and the eight EEG channels used in the current study is not sufficient for acceptable ICA artifact removal. Future studies attempting to conduct concurrent EEG-tDCS may benefit from the addition of a task during tDCS in order to facilitate discrimination between task-related activity and stimulation artifacts (Wirth et al., 2011; Roy et al., 2014; Soekadar et al., 2014). Additionally, increasing the number of EEG channels would improve the ability of ICA to perform artifact removal. However, a recent study suggests that the EEG artifacts induced by tDCS may be non-linear and that current artifact rejection methods may hence fail to fully remove these artifacts (Noury et al., 2016).

## Conclusion

Anodal tDCS of the left DLPFC using a high current density bi-frontal electrode montage resulted in detectable changes in resting-state EEG following stimulation, specifically an increase in power at lower frequencies with a peak of 7 Hz, and a decrease in power at higher frequencies above 20 Hz. Calculation of the mean EEG frequency revealed a generalized slowing of oscillations following active tDCS. However, similar changes in resting-state EEG were also observed following sham stimulation, which were lesser than after active tDCS, but nevertheless significant. In the sham condition changes in mean EEG frequency were correlated with changes in subjective arousal. The “online” effects of tDCS were not evaluated due to the extensive artifacts observed during stimulation. A task-related design—rather than resting-state EEG—may help to maintain a more constant physiological state and improve monitoring the effects of tDCS on cortical activity.

## Author Contributions

TWB, A-CM, DMM and CKL designed the study. A-CM acquired the data. TWB, SN analyzed the data and drafted the manuscript. TWB, SN, A-CM, DMM and CKL revised and approved the final manuscript.

## Conflict of Interest Statement

The authors declare that the research was conducted in the absence of any commercial or financial relationships that could be construed as a potential conflict of interest.
